# Republicans are flagged more often than Democrats for sharing misinformation on X’s Community Notes

**DOI:** 10.1073/pnas.2502053122

**Published:** 2025-06-16

**Authors:** Thomas Renault, Mohsen Mosleh, David G. Rand

**Affiliations:** ^a^Maison des Sciences Économiques, University Paris 1 Panthéon-Sorbonne, CNRS, Centre d’Economie Sorbonne, Paris 75013, France; ^b^Oxford Internet Institute, University of Oxford, Oxford OX1 3JS, United Kingdom; ^c^Sloan School of Management, Massachusetts Institute of Technology, Cambridge, MA 02138; ^d^Initiative on the Digital Economy, Massachusetts Institute of Technology, Cambridge, MA 02138; ^e^Department of Brain and Cognitive Sciences, Massachusetts Institute of Technology, Cambridge, MA 02139

**Keywords:** misinformation, social media, community notes, fact-checking

## Abstract

We use crowd-sourced assessments from X’s Community Notes program to examine whether there are partisan differences in the sharing of misleading information. Unlike previous studies, misleadingness here is determined by agreement across a diverse community of platform users, rather than by fact-checkers. We find that 2.3 times more posts by Republicans are flagged as misleading compared to posts by Democrats. These results are not base rate artifacts, as we find no meaningful overrepresentation of Republicans among X users. Our findings provide strong evidence of a partisan asymmetry in misinformation sharing which cannot be attributed to political bias on the part of raters, and indicate that Republicans will be sanctioned more than Democrats even if platforms transition from professional fact-checking to Community Notes.

Both Twitter (now X) and Meta have eschewed professional fact-checking in favor of user-based Community Notes programs because of claims that fact-checking programs are biased against Republicans. If Republicans share more false and misleading content than Democrats, however, even politically neutral and unbiased enforcement will preferentially sanction Republicans (1).

Indeed, a substantial body of research has found that Republicans share more misinformation on social media than Democrats (1–3). These studies, however, predominantly rely on evaluations by fact-checkers, journalists, and academics—and if these groups tend to have liberal leanings, the observed partisan asymmetry could simply reflect bias on the part of the evaluators rather than a true partisan difference in misinformation sharing. Furthermore, most prior research only examines posts containing URLs, which represent a small fraction of all social media content (4), and uses domain quality as a proxy of content accuracy, rather than actually evaluating specific claims. Conversely, survey studies examine hypothetical sharing of specific articles (5), but this method also introduces potential bias because the researchers select which headlines to include. Consequently, it remains unclear whether Republicans genuinely share more misleading content on social media.

Here, we address this issue by examining partisan asymmetries in crowd-sourced ratings from X’s Community Notes program. In Community Notes, users on X can flag posts as potentially misleading and write notes explaining why. Other users then vote on whether or not a given note is helpful. The platform uses an open-source “bridging algorithm” that requires agreement from users with diverse perspectives to determine whether a note is “helpful” (implying that the flagged post is misleading); see ref. 6 for details. Because they are based on the “vox populi,” crowdsourced methods like Community Notes are less susceptible to accusations of bias than centralized authorities like professional fact-checkers. If the tendency for Republicans to be more frequently sanctioned by social media companies is due to fact-checker bias, then we would not expect partisan differences in who is flagged on Community Notes.

## Results

**Fig. 1.**
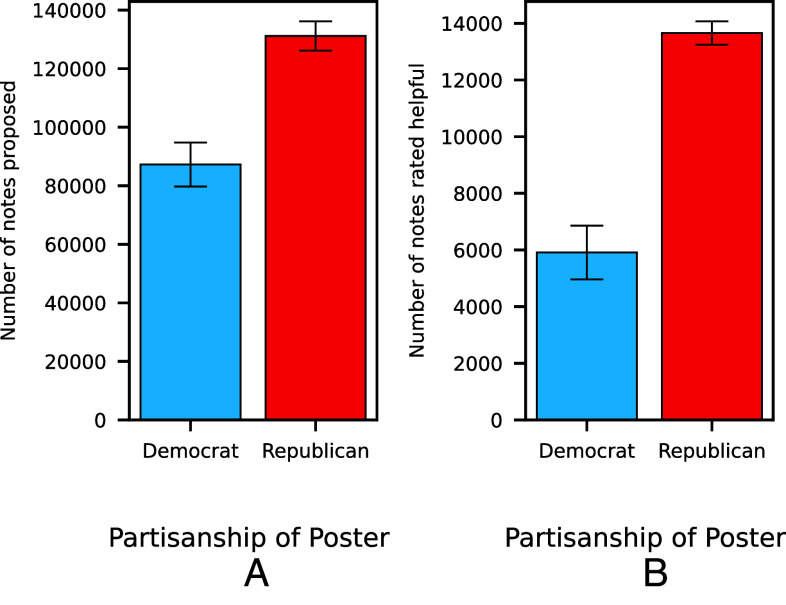
Posts by Republicans are flagged as potentially misleading much more often than posts by Democrats on X’s Community Notes. (*A*) Number of notes proposed on posts from Republicans versus Democrats. (*B*) Number of notes rated helpful on posts from Republicans versus Democrats.

We examine all English-language notes written between January 2023 and June 2024, and infer posters’ partisanship based on their social connections following the methodology proposed by Mosleh and Rand (7) and Barberá (8), and resolving classification disagreements between the two using a large language model applied to tweet content; see Methods and SI. Out of the 218,382 proposed Community Notes in our dataset, significantly more were proposed on posts by Republicans (60.05%) than posts by Democrats (39.95%) (α = 0.4078, 95% CI [0.288, 0.527], *P* < 0.001) (Fig. 1*A*).

However, just because a Community Note is proposed on a tweet does not mean that the tweet is misleading. For example, highly polarized discussions may attract notes simply due to ideological conflicts, regardless of the tweets’ actual accuracy. Thus, we use whether a note was rated helpful by the bridging algorithm—indicating that a diverse group of users agreed that the note was accurate—as a proxy for whether the flagged tweet was actually misleading. For instance, 1,011 Community Notes were proposed on tweets from Joe Biden (@JoeBiden) during our sample period, yet only 13 reached the helpful status (1.2% of the notes proposed).

Overall, we observe that 6.78% of notes proposed on tweets from Democrats are rated as helpful, compared to 10.41% of notes proposed on tweets from Republicans. Using a logistic regression that accounts for user-level characteristics (verified status, follower count, and total tweet volume) and the topic of the tweet, we find that being a Republican poster increases the likelihood of a proposed note achieving helpful status by 63.49% (β = 0.4916, 95% CI [0.383, 0.601], *P* < 0.001).

The partisan asymmetry is thus overall much starker among the 19,569 notes that were rated helpful. Here, 69.79% of flagged posts were written by Republicans (13,658 posts), compared to 30.21% written by Democrats (5,911 posts). In other words, 2.3 times as many posts by Republicans are flagged as misleading compared to posts by Democrats (α = 0.8375, 95% CI [0.673, 1.002], *P* < 0.001). (Fig. 1*B*). The partisan asymmetry remains highly significant when we infer political leaning using only the method from ref. 7 (1.7 times as many; α = 0.5159, 95% CI [0.328, 0.704], *P* < 0.001) or only the method from ref. 8 (2.1 times as many; α = 0.7525, 95% CI [0.596, 0.909], *P* < 0.001).

Using the topic classifier proposed by Chuai et al. (9), we find that 35.1% of the tweets with proposed notes pertain to *Politics*, 12.7% to *Science*, 7.8% to *Health*, 5.3% to the *Economy*, and the remaining 39.1% to various other topics. Notably, we find substantial partisan differences in the proportion of tweets rated as helpful across these topics. Focusing on notes rated as helpful by the bridging algorithm, Republican users are most overrepresented in misinformation sharing on *Health* (81.9% of flagged posts written by Republicans), followed by *Politics* (73.3%), *Science* (68.8%), *Other Topics* (66.9%), and the *Economy* (63.7%).

**Fig. 2.**
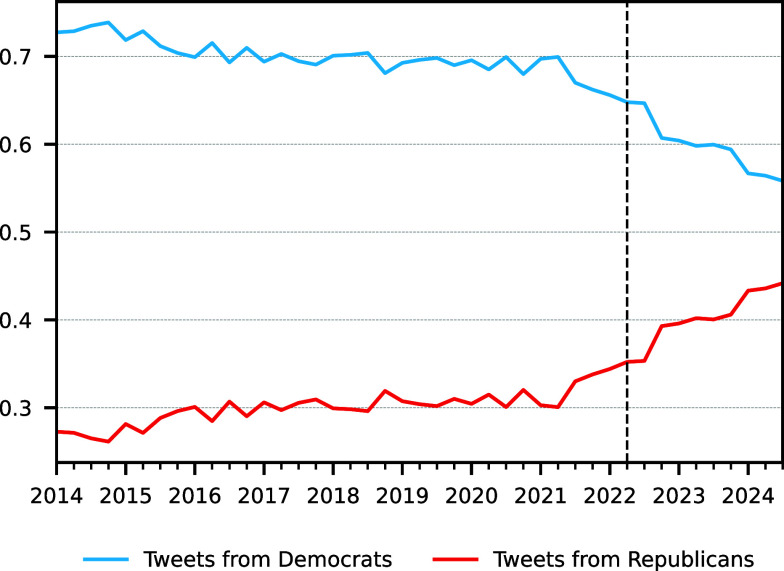
Democrats post more on X than Republicans. Shown is the fraction of a random sample of 474,394 posts including the word “the” that were posted by Democrats versus Republicans, with user partisanship estimated based on the accounts the user follows. The vertical line represents the acquisition of X (Twitter) by Elon Musk.

Together, these analyses show that the Community Notes program identifies substantially more misleading tweets from Republicans than Democrats. We now address two potential confounds. First, the difference in flagging we observe could reflect an overrepresentation of Republicans on Twitter, which might increase the likelihood of Republicans being flagged. To evaluate this possibility, we use X’s Pro API to extract a random sample of tweets in English containing the stopword “the” (474,394 tweets from January 1st, 2014, to November 1st 2024). For each tweet, we determine the political leaning of the tweet’s creator using ref. 7. We find that while the percentage of Republican users has increased considerably since Elon Musk’s takeover, the proportion of Democrats is still higher than that of Republicans (Fig. 2).

Second, our results might reflect an overrepresentation of Democrats participating in Community Notes, which also might increase the likelihood of Republicans being flagged. Given evidence of counterpartisan flagging on Community Notes (6), an overrepresentation of Democratic flaggers could create a partisan imbalance in *proposed* Notes (Fig. 1*A*). However, Community Notes’ bridging algorithm—which requires agreement from users who typically disagree in order to rate a Note as helpful—would tend to reduce such an imbalance when examining helpful Notes. Yet we observe the opposite pattern, wherein Republicans are preferentially flagged to a much greater extent among helpful Notes (Fig. 1*B*). This suggests that the pattern cannot be attributed to the partisan makeup of Community Notes contributors.

## Discussion

Our findings provide strong evidence of a partisan asymmetry in misinformation sharing which cannot be attributed to political bias on the part of fact-checkers or academic researchers. Even on Elon Musk’s X, the user-based Community Notes program flags posts by Republicans as misleading much more often than posts by Democrats. This undercuts the logic offered by Musk and Mark Zuckerberg for eliminating fact-checkers on X and Meta, respectively, namely that fact-checkers are biased against Republicans. Our results demonstrate that even Community Notes, where diverse groups of users have to agree in order for posts to be flagged, finds far more misleading content being posted by Republicans compared to Democrats.

## Materials and Methods

We use the full open-source dataset of Twitter’s (X’s) Community Notes, focusing specifically on notes flagged posts as “misinformed or potentially misleading” that were written in English and targeting English-language tweets posted between January 2023 and June 2024. Our study was deemed exempt by the MIT Committee On the Use of Humans as Experimental Subjects, exempt ID E-6711. For each proposed note, we track whether it has garnered sufficient positive ratings from a diverse audience to be published (see ref. 10 for a description of the algorithm used to compute note helpfulness scores). Once deemed helpful, Community Notes are displayed on Twitter beneath the relevant tweets, helping to flag tweets with potentially false or misleading information.

We infer users’ political leaning based on their social connections, following the methodologies from refs. 7 and 8. Using ref. 7, we assign a partisan score to users who follow at least one political elite account, classifying users with a score >0 as Republican and <0 as Democrat. Separately, we use the statistical models from ref. 8 to generate a continuous ideology score ranging from −2.5 (strongly liberal) to 2.5 (strongly conservative), and based on the results presented in ref. 6 we classify users with scores >1 as Republican and scores ≤1 as Democrat. When classifications from refs. 7 and 8 disagree, or if one is unavailable, we apply a large language model to analyze the user’s 500 most recent tweets and infer political leaning using GPT-4o mini (*SI Appendix*). Users are included in the analysis only if they receive consistent classifications from at least two of the three methods (refs. 7 and 8, and GPT-4o). Our final dataset includes 218,382 Community Notes, covering 162,228 tweets from 39,140 unique users.

## Supplementary Material

Appendix 01 (PDF)

## Data Availability

The code and a dataset of dehydrated tweets, compliant with X’s Terms of Service, are publicly available at https://github.com/trenault/CommunityNotes/ (11). Researchers can hydrate the dataset by registering for an X Developer Account at https://developer.x.com/en (12).

## References

[1] M. Mosleh, Q. Yang, T. Zaman, G. Pennycook, D. G. Rand, Differences in misinformation sharing can lead to politically asymmetric sanctions. Nature **634**, 609–616 (2024). 39358507 10.1038/s41586-024-07942-8 PMC11485227

[2] N. Grinberg, K. Joseph, L. Friedland, B. Swire-Thompson, D. Lazer, Fake news on Twitter during the 2016 US presidential election. Science **363**, 374–378 (2019). 30679368 10.1126/science.aau2706

[3] S. González-Bailón , Asymmetric ideological segregation in exposure to political news on Facebook. Science **381**, 392–398 (2023). 37499003 10.1126/science.ade7138

[4] A. Aljebreen, W. Meng, E. Dragut, “Segmentation of tweets with URLs and its applications to sentiment analysis” in Proceedings of the AAAI Conference on Artificial Intelligence (2021), vol. **35**, no. 14, pp. 12480–12488.

[5] G. Pennycook, J. Binnendyk, C. Newton, D. G. Rand, A practical guide to doing behavioral research on fake news and misinformation. Collabra **7**, 25293 (2021).

[6] J. Allen, C. Martel, D. G. Rand, “Birds of a feather don’t fact-check each other: Partisanship and the evaluation of news in Twitter’s Birdwatch crowdsourced fact-checking program” in Proceedings of the 2022 CHI Conference on Human Factors in Computing Systems (2022), pp. 1–19.

[7] M. Mosleh, D. G. Rand, Measuring exposure to misinformation from political elites on Twitter. Nat. Commun. **13**, 7144 (2022). 36414634 10.1038/s41467-022-34769-6 PMC9681735

[8] P. Barberá, Birds of the same feather tweet together: Bayesian ideal point estimation using Twitter data. Polit. Anal. **23**, 76–91 (2015).

[9] Y. Chuai , Community-based fact-checking reduces the spread of misleading posts on social media. arXiv [Preprint] (2024). 10.48550/arXiv.2409.08781 (Accessed 5 May 2025).

[10] S. Wojcik , Birdwatch: Crowd wisdom and bridging algorithms can inform understanding and reduce the spread of misinformation. arXiv [Preprint] (2022). 10.48550/arXiv.2210.15723 (Accessed 5 May 2025).

[11] T. Renauld, CommunityNotes dataset. GitHub. https://github.com/trenault/CommunityNotes. Accessed 4 April 2025.

[12] X Corp, X Developer Platform. X Corp. https://developer.x.com/en. Accessed 5 May 2025.

