# Supplementary material for: Republicans are flagged more often than Democrats for sharing misinformation on X’s Community Notes
Source: Proc Natl Acad Sci U S A. 2025 Jun 16;122(25):e2502053122. doi: 10.1073/pnas.2502053122 (PMC12207429; doi:10.1073/pnas.2502053122)
Supplement: Supplementary file 1 — Appendix 01 (PDF) [file pnas.2502053122.sapp.pdf]

Supporting information

for

Republicans are flagged more often than Democrats for sharing misinformation on X's Community Notes

As outlined in the main text, we infer each user's political leaning using three distinct methods: two network-based approaches relying on users' social connections following the methodologies proposed by [1] and [2], and a content-based classification using a large language model (LLM).

Using [1], we assign a partisan score to users who follow at least one political elite account, classifying users with a score > 0 as Republican and < 0 as Democrat. Separately, we use the statistical models from [2] to generate a continuous ideology score ranging from -2.5 (strongly liberal) to 2.5 (strongly conservative), classifying users with scores > 1 as Republican and scores ≤ 1 as Democrat. For [2], we adopt a threshold of 1 for identifying Republican users rather than the default 0.5 in order to maximize consistency between the outputs of [1] and [2] and to remain conservative (i.e, the over-representation of Republicans is even larger if we use the threshold of 0.5).

Missing classifications can arise due to differences in the coverage of each method. The [1] model depends on users' connections to political elites as of 2022, while [2] is recomputed using follower data from March 2025. As a result, users who created accounts after 2022 or who do not follow any political elites included in [1] are not assigned a score by this method. Similarly, users may be excluded from [2] if they do not follow any elite accounts used in that model.

To address cases where the two network-based approaches disagree or where one classification is missing, we apply a third method based on content analysis using an LLM. For each user, we collect their 500 most recent tweets and classify them using the GPT-4o mini model with the following prompt:

You are an AI assistant that must only classify a user based on the provided list of political leaning. Rules: - Do not generate, infer, or suggest any responses outside the list. - Your response must be exactly one of the provided answers. - Do not provide explanations, justifications, or additional context. Task: Question: Based on the following tweets, what is the most likely political leaning of the person who posted them? Tweets: [Last 500 tweets here]. Potential Answers: - democrat - republican . Your response should be only one of these words.

The agreement rate between [1] and [2] is 87.04%. The agreement rate between the LLM-based classification and the [1] method is 82.06% (when [2] is missing) and 78.38% with the [2] method (when [1] is missing).

The sample includes 169,270 Community Notes when using only [1], 229,393 when using only [2], and 218,382 when combining [1], [2], and the LLM (assigning the majority label as the final classification when at least two of the three methods agree).

References

[1] Mohsen Mosleh and David G Rand. Measuring exposure to misinformation from political elites on Twitter. *Nature Communications*, 13(1):7144, 2022.

[2] Pablo Barberá. Birds of the same feather tweet together: Bayesian ideal point estimation using twitter data. *Political analysis*, 23(1):76–91, 2015.
